# Prognostic factors in patients with HBV-related hepatocellular carcinoma following hepatic resection

**DOI:** 10.1186/s13027-018-0192-7

**Published:** 2018-06-08

**Authors:** Narongsak Rungsakulkij, Wikran Suragul, Somkit Mingphruedhi, Pongsatorn Tangtawee, Paramin Muangkaew, Suraida Aeesoa

**Affiliations:** Department of Surgery, Faculty of Medicine, Ramathibodi Hospital, Mahidol University, 270 Praram VI Road, Ratchathewi, Bangkok, 10400 Thailand

**Keywords:** Alpha-fetoprotein, Hepatitis B virus, Hepatocellular carcinoma, Risk factors, Survival rate

## Abstract

**Background:**

To analyze prognostic factors following hepatic resection in patients with HBV-related hepatocellular carcinoma.

**Methods:**

We retrospectively analyzed 217 patients with HBV-related hepatocellular carcinoma who underwent hepatic resection at our hospital between January 2006 and December 2015. Disease-free survival and overall survival rates were analyzed using the Kaplan–Meier method and the log-rank test. The association between recurrence and survival and various clinicopathological factors, including serum alpha-fetoprotein (AFP) level, platelet count, platelet-to-lymphocyte ratio, neutrophil-to-lymphocyte ratio, antiplatelet therapy, antiviral therapy, hepatitis C virus infection, and tumor-related characteristics, were assessed using univariate and multivariate logistic regression analysis.

**Results:**

The 1-, 3-, and 5-year overall survival rates were 91, 84, and 79%, respectively, and the recurrence-free survival rates were 72, 51, and 44%, respectively. High post-operative AFP level (hazard ratio [HR] 1.112, 95% confidence interval [CI]: 1.02–1.21, *P* = 0.007), multiple tumors (HR 1.991, 95% CI: 1.11–3.56, *P* = 0.021), and no antiviral treatment (HR 1.823, 95% CI: 1.07–3.09, *P* = 0.026) were independent risk factors for recurrence. High post-operative AFP level (HR 1.222, 95% CI: 1.09–1.36, *P* < 0.001), multiple tumors (HR 2.715, 95% CI: 1.05–7.02, *P* = 0.039), and recurrence (HR 12.824, 95% CI: 1.68–97.86, *P* = 0.014) were independent risk factors for mortality. No other factors analyzed were associated with outcomes in this patient cohort.

**Conclusions:**

High post-operative serum alpha-fetoprotein level and multiple tumors, but not inflammatory factors, were risk factors for poor prognosis in HBV-related hepatocellular carcinoma patients after resection.

## Background

Hepatocellular carcinoma (HCC) is the most common type of primary liver cancer worldwide [1]. The Eastern Asia and sub-Saharan Africa are the highest areas in hepatitis B virus (HBV) related HCC [2]. In Thailand, HCC is most frequently caused by chronic HBV infection [3, 4]. Surgical resection is potentially curative for early-stage disease if liver functional reserve is adequate [5], but its outcome in HBV-related HCC patients is generally poor [6]. Cirrhosis, chronic hepatitis [7, 8], and chronic HBV infection are considered to be poor prognostic factors following hepatic resection in HCC patients [9].

Inflammation is a key contributor to the pathogenesis of HCC in patients with chronic HBV infection [10–12]. Many studies have investigated the utility of inflammatory factors and indices as prognostic markers for HBV-related HCC patients following hepatic resection; however, the results are controversial [13–19]. Recent reports suggest that platelets play a major role in the pathogenesis of HCC in HBV-infected patients [20, 21]. Indeed, antiplatelet therapy reduces the incidence of HCC in an HBV-infected mouse model [22]. In addition, Lee et al. reported that HBV-related HCC patients receiving antiplatelet therapy showed better recurrence-free and overall survival after liver resection than untreated patients [23]. Given these observations, we investigated the prognostic value of platelet counts, antiplatelet therapy, inflammatory indices, and various tumor-related characteristics in patients with HBV-related HCC following hepatic resection.

## Methods

A total of 387 consecutive patients underwent liver resection and had pathologically proven HCC at the Department of Surgery, Ramathibodi Hospital, Mahidol University, Bangkok, Thailand between January 2006 and December 2015. All patients were followed-up until December 2017. Of these, we retrospectively analyzed data from the 217 patients with HBV-related HCC. The patients who had HDV co-infection were excluded from the study. All patients underwent preoperative cross-sectional dynamic imaging using either triple-phase CT or magnetic resonance imaging (MRI). Routine blood examinations included complete blood count, coagulogram, liver and kidney function tests, and preoperative serum alpha-fetoprotein (AFP) level. The serum AFP level are measured by electrochemiluminescence immunoassay method, AFP ELISA reagent Roche Elecsys®, Roche Diagnostics USA, Indiana, United State. The neutrophil-to-lymphocyte ratio and platelet-to-lymphocyte ratio were calculated. The prognostic nutritional index was calculated as ([albumin {g/L} + 0.005] × [total lymphocyte count {/μL}]). A preoperative indocyanine green retention test at 15 min (ICG-R15) was performed. The Makuuchi criteria are used for patient selection for curative resection in our center [24]. The extent of liver resection was based on the patient’s liver functional reserve as assessed mainly by the Makuuchi criteria, including preoperative ascites volume, Child–Pugh score, ICG-R15 value, and, occasionally, volumetric CT analysis. Liver cirrhosis was defined by the macro or micro nodular surface of the liver intraoperatively.

Pathological specimens were reviewed by a pathologist to confirm the diagnosis of HCC. Patients with combined cholangiocarcinoma and other malignancies were excluded from this study. Microvascular invasion was defined as the presence of tumor cells in the microvasculature. Clinical and pathologic staging was performed according to the American Joint Committee on Cancer staging manual 7th edition [25].

Patients were followed up in outpatient clinics every 3 or 4 months after surgery and routinely underwent imaging studies (ultrasonography, CT, MRI) and blood examinations. Post-operative serum AFP levels were measured within 90 days after hepatic resection. Recurrent disease was defined as the presence of new tumors found by imaging (CT or MRI) during the follow-up period.

### Statistical analyses

Patient characteristics with continuous variables were compared by Student’s t-test, and categorical variables were compared with χ2 or Fisher’s exact test. A *P* value of < 0.05 was considered statistically significant. The potential risk factors were analyzed by univariate and multivariate methods using a Cox regression model. Independent risk factors were expressed as hazard ratios (HR) with 95% confidence intervals (CI). Survival analysis was performed using the Kaplan–Meier method and evaluated by the log-rank test. The cut-off value for post-hepatectomy serum AFP level was determined by receiver operating characteristic (ROC) curve analysis with most significance in predicting tumor recurrence after hepatectomy.

## Results

### Patient characteristics and perioperative status

Of the 387 consecutive patients who underwent curative resection for HCC from January 2006 to December 2015, 217 (56.0%) had HBV-related HCC and were evaluated here. The clinicopathological characteristics of this cohort are summarized in Table 1.

**Table 1:** Clinicopathological features of patients with HBV-related hepatocellular carcinoma

Characteristic	Value
Gender, *n* (%) (total cohort *n* = 217)	
male	100 (46.08)
female	117 (53.92)
Age (years), mean ± sd	56.12 (9.78)
HBsAg, *n* (%)	
negative	16 (7.37)
positive	201 (92.62)
HBeAg, *n* (%), *n* = 119	
negative	85 (71.43)
positive	34 (28.57)
HBV DNA, n (%), *n* = 103	
negative	41 (39.81)
positive	62 (60.19)
HCV, *n* (%)	
no	210 (96.77)
yes	7 (3.23)
Platelets × 10^3^ (mm3), median (range)	190.5 (57, 568)
AFP-pre (ng/mL), median (range), *n =* 185	16.8 (0.89, 82,392)
AFP-post (ng/mL), median (range), *n =* 125	3.48 (0.83, 19,629)
Tumor size (cm), median (range), *n =* 216	4.5 (0.5, 26.5)
< 5	120 (55.56)
≥ 5	96 (44.44)
Number of tumors, *n* (%)	
solitary	166 (77.57)
multiple	48 (22.43)
Microvascular invasion, *n* (%)	
no	170 (79.44)
yes	44 (20.56)
Stage, *n* (%)	
I	138 (63.59)
II or higher	79 (36.41)
Resection margin, *n* (%), *n =* 185	
free margin	176 (95.14)
positive margin	9 (4.86)
Operation type, *n* (%)	
non-anatomical	129 (59.45)
anatomical	88 (40.55)
Preoperative neoadjuvant, *n* (%), *n =* 164	
no	92 (56.10)
yes	72 (43.90)
Platelet-to-lymphocyte ratio, median (range), *n =* 203	101.8 (30.9, 432.8)
Prognostic nutritional index, mean ± sd *n =* 206	95.18 (40.21)
Neutrophil-to-lymphocyte ratio, median (range), *n =* 201	1.77 (0.33, 10.62)
Antiviral treatment	
no	65 (29.95)
yes	152 (70.05)
Antiviral drug, *n* (%)	
Adefovir	7 (3.23)
Lamivudine	125 (57.60)
Tenofovir	44 (20.28)
Entecavir	20 (9.22)
Antiplatelet treatment (ASA + Clopidogrel)	
no	199 (91.71)
yes	18 (8.29)
Recurrence, *n* (%)	
no	113 (52.07)
yes	104 (47.93)
Follow-up time (months), median (range)	36.33 (0.23, 149.07)

*AFP* alpha-fetoprotein, *ASA* aspirin, *HCV* hepatitis C virus, *sd* standard deviation

### Risk factors associated with disease recurrence

A comparison between patients with and without disease recurrence is shown in Table 2. The recurrence rate following resection was 47.9% (104/217). Compared with the non-recurrence group, the recurrence group had a higher post-operative AFP level (2.8 vs 3.8 ng/mL, *P* = 0.045), was more likely to have multiple tumors (32 vs 16 patients, *P* = 0.004), and was less likely to have received preoperative neoadjuvant treatment (48/92 vs 26/72 patients, *P* = 0.04). Univariate analysis (Table 3) identified the following factors as significantly associated with disease recurrence: post-operative AFP level (HR 1.112, 95% CI: 1.02–1.21, *P* = 0.012), tumor size (HR 1.061, 95% CI: 1.01–1.11, *P* = 0.013), multiple tumors (HR 1.881, 95% CI: 1.23–2.86, *P* = 0.003), microvascular invasion (HR 1.645, 95% CI: 1.02–2.63, *P* = 0.037), stage II or higher (HR 1.553, 95% CI 1.04–2.31, *P* = 0.031), and no antiviral treatment (HR 1.519, 95% CI: 1.01–2.28, *P* = 0.045). In multivariate analysis (Table 3), post-operative AFP (HR 1.112, 95% CI: 1.02–1.21, *P* = 0.007), multiple tumors (HR 1.991, 95% CI: 1.11–3.56, *P* = 0.021), and no antiviral treatment (HR 1.823, 95% CI: 1.07–3.09, *P* = 0.026) remained independent risk factors for recurrence.

**Table 2:** Clinicopathological features of patients in the non-recurrence and recurrence groups

Characteristic	Non-Recurrence (*n =* 113)	Recurrence (*n =* 104)	*P* value
Gender, *n* (%) (total cohort *n* = 217)			
male	49 (43.36)	51 (49.04)	0.402
female	64 (56.64)	53 (50.96)	
Age (years), mean ± sd	56.46 (10.60)	55.76 (8.86)	0.604
HCV, *n* (%)			
no	111 (98.23)	99 (95.19)	0.264
yes	2 (1.77)	5 (4.81)	
Platelets × 103, median (range), *n =* 384	198.5 (57, 465)	179.5 (76, 568)	0.068
AFP-pre (ng/mL), median (range), *n =* 325	15.2 (0.89, 60,500)	17.03 (1.1, 82,392)	0.572
AFP-post (ng/mL), median (range), *n =* 226	2.8 (0.83, 5271)	3.8 (0.9, 19,629)	*0.045*
Tumor size (cm), median (range), *n =* 386	4.3 (0.6, 26.5)	5 (0.5, 18)	0.511
< 5	63 (55.75)	57 (55.34)	0.951
≥ 5	50 (44.25)	46 (44.66)	
Number of tumors, *n* (%), *n =* 382			
solitary	94 (85.45)	72 (69.23)	*0.004*
multiple	16 (14.55)	32 (30.77)	
Microvascular invasion, *n* (%), *n =* 382			
no	89 (80.91)	81 (77.88)	0.584
yes	21 (19.09)	23 (22.12)	
Stage, *n* (%)			
I	77 (68.14)	61 (58.65)	0.147
II or higher	36 (31.86)	43 (41.35)	
Resection margin, *n* (%), *n =* 325			
free margin	89 (94.68)	87 (95.60)	0.999
positive margin	5 (5.32)	4 (4.40)	
Operation type, *n* (%)			
non-anatomical	69 (61.06)	60 (57.69)	0.614
anatomical	44 (38.94)	44 (42.31)	
Preoperative neoadjuvant, *n* (%), *n =* 289			
no	44 (48.89)	48 (64.86)	*0.040*
yes	46 (51.11)	26 (35.14)	
Platelet-to-lymphocyte ratio, median (range), *n =* 365	106.6 (46.3, 432.8)	91.2 (30.9, 290.7)	0.128
Prognostic nutritional index, median (range), *n =* 370	89.12 (0.34, 265.26)	91.9 (0.41, 245.02)	0.764
Neutrophil-to-lymphocyte ratio, median (range), *n =* 361	1.78 (0.67, 8.11)	1.76 (0.33, 10.62)	0.770
Antiviral treatment			
no	30 (26.55)	35 (33.65)	0.254
yes	83 (73.45)	69 (66.35)	
Antiviral drug			
Adefovir	4 (3.54)	3 (2.88)	0.999
Lamivudine	66 (58.41)	59 (56.73)	0.254
Tenofovir	28 (25.66)	15 (14.42)	0.021
Entecavir	10 (8.85)	10 (9.62)	0.846
Antiplatelet treatment (ASA + Clopidogrel)			
no	103 (91.15)	96 (92.31)	0.757
yes	10 (8.85)	8 (7.69)	

*AFP* alpha-fetoprotein, *ASA* aspirin, *HCV* hepatitis C virus, *sd* standard deviation

NOTE. Italic font indicates statistical significance

**Table 3:** Univariate and multivariate analysis of factors associated with recurrence

	Univariate		Multivariate	
	HR (95% CI)	*P* value	HR (95% CI)	*P* value
Gender (male)				
female	0.894 (0.60–1.32)	0.574		
Age (years)	0.996 (0.97–1.02)	0.719		
HCV (no)				
yes	1.473 (0.59–3.62)	0.399		
Platelets × 103 (mm3)	0.987 (0.96–1.01)	0.367		
AFP-pre (ng/mL)	0.996 (0.97–1.01)	0.665		
AFP-post (ng/mL)	1.112 (1.02–1.21)	*0.012*	1.129 (1.04–1.23)	*0.005*
Tumor size (< 5 cm)	1.061 (1.01–1.11)	*0.013*		
≥ 5 cm	1.345 (0.90–1.99)	0.139		
Number of tumors (solitary)				
multiple	1.881 (1.23–2.86)	*0.003*	1.973 (1.15–3.38)	*0.013*
Microvascular invasion (no)				
yes	1.645 (1.02–2.63)	*0.037*		
Stage (I)				
II or higher	1.553 (1.04–2.31)	*0.031*		
Resection margin (free margin)				
positive margin	0.977 (0.35–2.66)	0.964		
Operation type (anatomical)				
non-anatomical	0.708 (0.47–1.05)	0.085		
Preoperative neoadjuvant (no)				
yes	0.828 (0.51–1.34)	0.450		
Platelet-to-lymphocyte ratio	0.913 (0.61–1.34)	0.648		
Prognostic nutritional index	0.959 (0.56–1.61)	0.875		
Neutrophil-to-lymphocyte ratio	1.052 (0.89–1.23)	0.535		
Antiviral treatment				
no	1.519 (1.01–2.28)	*0.045*	1.823 (1.07–3.09)	*0.026*
Antiplatelet treatment (ASA + Clopidogrel)				
no	1.018 (0.49–2.09)	0.961		

* AFP* alpha-fetoprotein, *ASA* aspirin, *CI* confidence interval, *HR* hazard ratio, *HCV* hepatitis C virus

NOTE. Italic font indicates statistical significance

### Risk factors associated with mortality

Table 4 shows the comparison of survivors and non-survivors. The survival rate of HBV-related HCC patients following hepatectomy was 82.5% (179/217). Compared with the survivor group, non-survivors had significantly higher pre- and post-operative AFP levels (115 vs 14.2 ng/mL, *P* = 0.018 and 13.11 vs 2.8 ng/mL, *P* < 0.001, respectively) and were more likely to have multiple tumors than a solitary tumor (14/48 vs 23/166 patients, *P* = 0.013). Patients undergoing anatomical resection also had a higher mortality rate than those undergoing other operations (22/88 vs 16/129, *P* = 0.017). As shown in Table 5, univariate analysis identified the following factors as significantly associated with survival: post-operative AFP level (HR 1.218, 95% CI: 1.10–1.35, *P* < 0.001), tumor size ≥5 cm (HR 1.679, 95% CI: 1.01–2.77, *P* = 0.044), multiple tumors (HR 2.300 95% CI: 1.18–4.47, *P* = 0.014), anatomical resection (HR 2.443, 95% CI: 1.28–4.65, *P* = 0.007), no antiviral treatment (HR 0.482, 95% CI: 0.25–0.92, *P* = 0.027), and recurrence (HR 2.940, 95% CI: 1.40–6.05, *P* = 0.003). In multivariate analysis, post-operative AFP (HR 1.222, 95% CI: 1.09–1.36, *P* < 0.001), multiple tumors (HR 2.715, 95% CI: 1.05–7.02, *P* = 0.039), and recurrence (HR 12.824, 95% CI: 1.68–97.86, *P* = 0.014) were independent risk factors for death (Table 5).

**Table 4:** Comparison of clinicopathological features of survivors and non-survivors

Characteristic	Alive (*n =* 179)	Dead (*n =* 38)	*P* value
Gender, *n* (%)			
male	76 (42.46)	24 (63.16)	*0.020*
female	103 (57.54)	14 (36.84)	
Age (years), mean ± sd	56.03 (9.44)	56.60 (11.39)	0.742
HCV, *n* (%)			
no	172 (96.09)	38 (100)	0.609
yes	7 (3.91)	0	
Platelets ×103 (mm3), median (range)	192 (57, 568)	185 (91, 332)	0.485
AFP-pre (ng/mL), median (range), *n =* 185	14.2 (0.89, 82,392)	115 (1.85, 60,500)	*0.018*
AFP-post (ng/mL), median (range), *n =* 125	2.8 (0.83, 5271)	13.11 (1.19, 19,629)	*0.0003*
Tumor size (cm), median (range), *n =* 216	4.3 (0.5, 26.5)	5.5 (2, 17)	0.066
< 5	103 (57.54)	17 (45.95)	0.196
≥ 5	76 (42.46)	20 (54.05)	
Number of tumors, *n* (%)			
solitary	143 (80.79)	23 (62.16)	*0.013*
multiple	34 (19.21)	14 (37.84)	
Microvascular invasion, *n* (%)			
no	141 (79.66)	29 (78.38)	0.861
yes	36 (20.34)	8 (21.62)	
Stage, *n* (%)			
I	110 (61.45)	28 (73.68)	0.155
II or higher	69 (38.55)	10 (26.32)	
Resection margin, *n* (%), *n =* 185			
free margin	144 (96.00)	32 (91.43)	0.375
positive margin	6 (4.00)	3 (8.57)	
Operation type, *n* (%)			
non-anatomical	113 (63.13)	16 (42.11)	*0.017*
anatomical	66 (36.87)	22 (57.89)	
Preoperative neoadjuvant, *n* (%) *n =* 164			
no	71 (53.79)	21 (65.63)	0.226
yes	61 (46.21)	11 (34.38)	
Platelet-to-lymphocyte ratio, median (range), *n =* 203	101.6 (30.9, 432.8)	107.1 (51.0, 258.9)	0.339
Prognostic nutritional index, mean ± sd, *n =* 206	97.35 (41.10)	84.21 (33.78)	0.082
Neutrophil-to-lymphocyte ratio, median (range), *n =* 201	1.73 (0.33, 10.62)	2 (0.73, 4.41)	0.298
Antiviral treatment			
no	49 (27.37)	16 (42.11)	0.072
yes	130 (72.63)	22 (57.89)	
Antiplatelet treatment (ASA + Clopidogrel)			
no	163 (91.06)	36 (94.74)	0.746
yes	16 (8.94)	2 (5.26)	
Recurrence *n* (%)			
no	103 (57.54)	10 (26.32)	*0.000*
yes	76 (42.46)	28 (73.68)	

*AFP* alpha-fetoprotein, *ASA* aspirin, *HCV* hepatitis C virus, microvascular invasion, *sd* standard deviation

NOTE. Italic font indicates statistical significance

**Table 5:** Univariate and multivariate analysis of factors associated with overall survival

	Univariate		Multivariate	
	HR (95% CI)	*P* value	HR (95% CI)	*P* value
Gender (male)				
female	0.552 (0.28–1.07)	0.080		
Age (years)	1.002 (0.96–1.04)	0.890		
HCV (no)				
yes	–			
Platelets × 103 (mm3)	0.999 (0.99–1.01)	0.829		
AFP-pre (ng/mL)	1.011 (0.99–1.03)	0.300		
AFP-post (ng/mL)	1.218 (1.10–1.35)	*0.000*	1.206 (1.08–1.34)	*0.000*
Tumor size (< 5 cm)	1.052 (0.99–1.12)	0.091		
≥ 5 cm.	1.679 (1.01–2.77)	*0.044*		
Number of tumors (solitary)				
multiple	2.300 (1.18–4.47)	*0.014*	2.715 (1.05–7.02)	*0.039*
Microvascular invasion (no)				
yes	1.598 (0.72–3.54)	0.249		
Stage (I)				
II or higher	0.737 (0.35–1.53)	0.415		
Resection margin (free margin)				
positive margin	2.140 (0.65–7.05)	0.211		
Operation type (anatomical)				
non-anatomical	0.409 (0.21–0.78)	*0.007*		
Preoperative neoadjuvant (no)				
yes	0.958 (0.45–2.01)	0.910		
Platelet-to-lymphocyte ratio	1.003 (0.99–1.01)	0.195		
Prognostic nutritional index	0.991 (0.98–1.00)	0.065		
Neutrophil-to-lymphocyte ratio	1.070 (0.82–1.39)	0.621		
Antiviral treatment				
no	0.482 (0.25–0.92)	*0.027*		
Antiplatelet treatment (ASA + Clopidogrel)				
no	1.542 (0.37–6.41)	0.551		
Recurrence (no)				
yes	2.940 (1.42–6.05)	*0.003*	12.824 (1.68–97.86)	*0.014*

*AFP* alpha-fetoprotein, *ASA* aspirin, *HCV* hepatitis C virus, *sd* standard deviation

NOTE. Italic font indicates statistical significance

### Overall survival and recurrence-free survival analysis

**Fig. 1:**
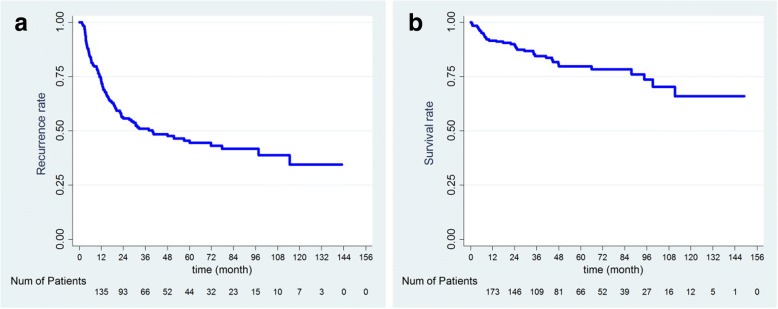
Kaplan–Meier survival analysis of HBV-related hepatocellular carcinoma following hepatic resection. **a**, overall recurrence; **b**, overall survival

**Fig. 2:**
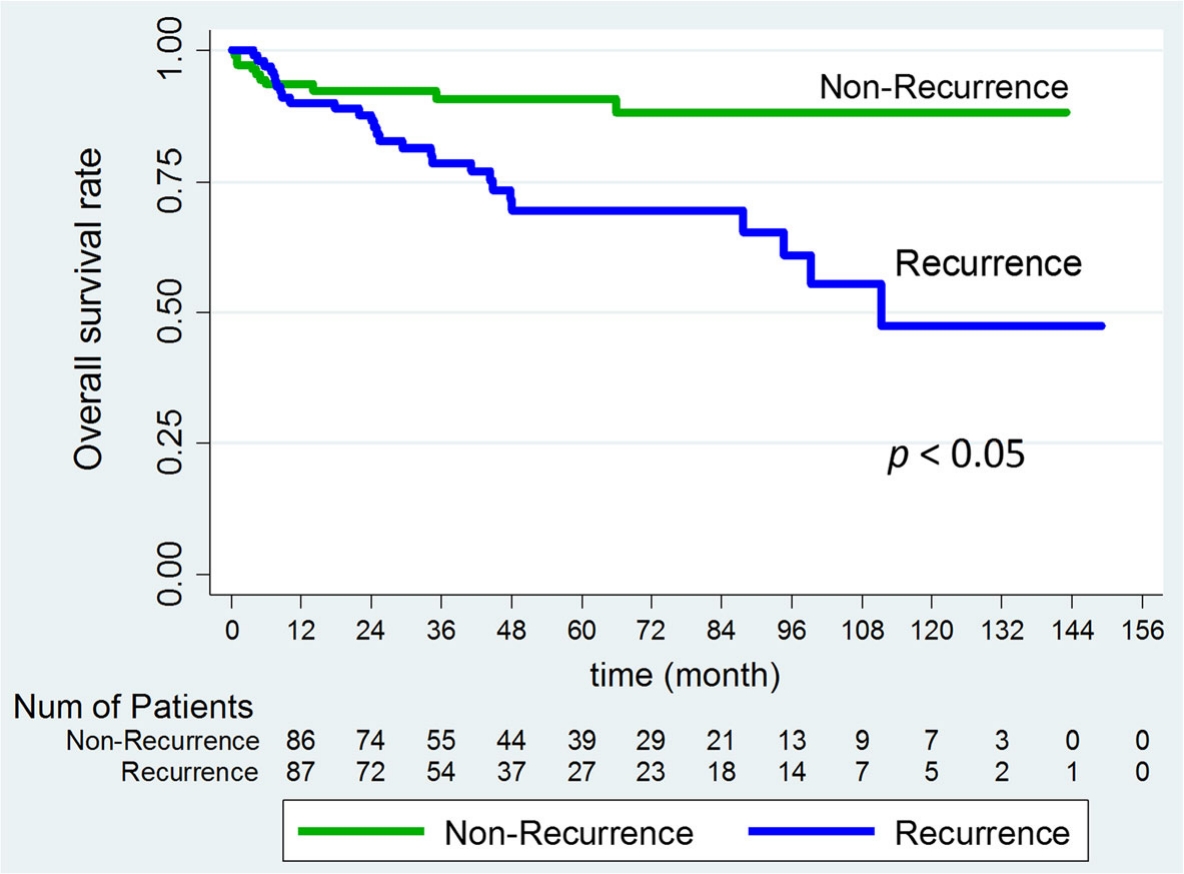
Kaplan–Meier survival analysis of the recurrence and non-recurrence groups

**Fig. 3:**
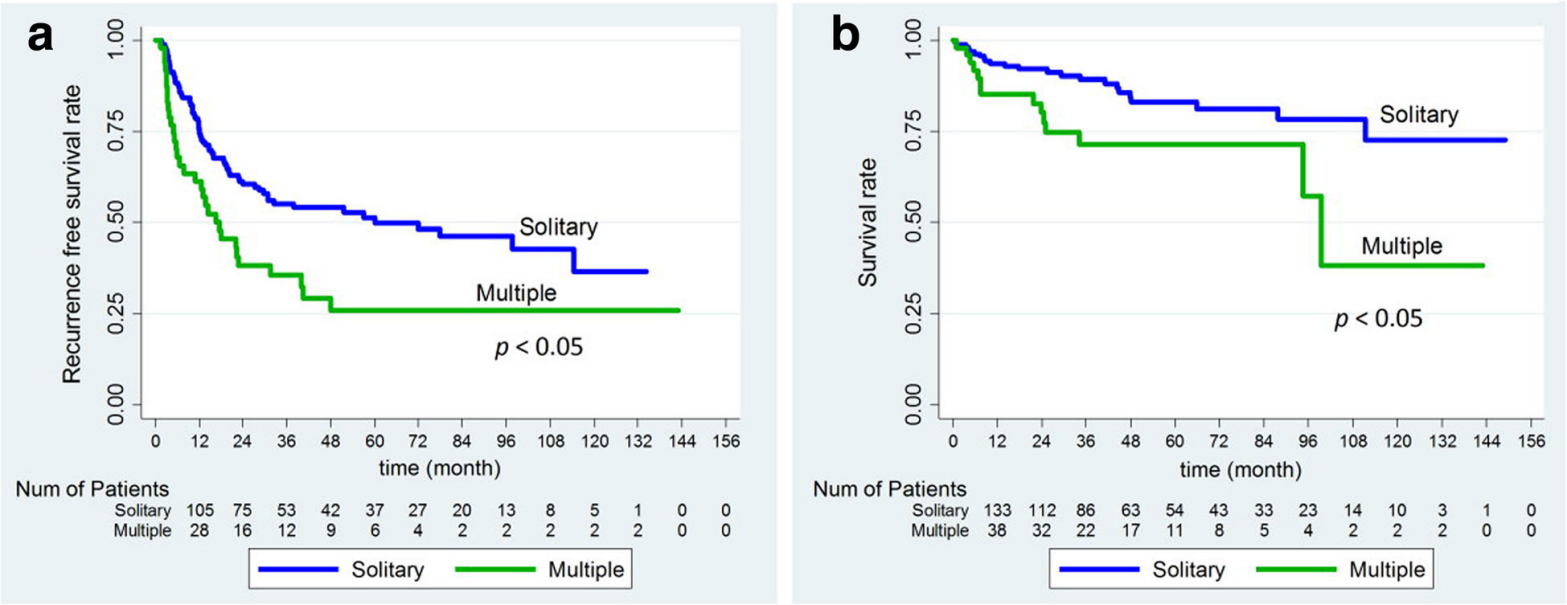
Kaplan–Meier survival analysis of patients with solitary and multiple tumors. **a**, recurrence-free survival; **b**, overall survival

The Kaplan–Meier analysis curves for recurrence-free survival (RFS) and overall survival (OS) of all patients are shown in Fig. 1. The overall 1-, 3-, and 5-year overall survival rates were 91, 84, and 79%, respectively, and the RFS rates were 72, 51, and 44%, respectively. As expected, OS was significantly poorer for patients with recurrent compared with non-recurrent disease (Fig. 2). In addition, patients with multiple tumors had poorer OS and RFS than patients with solitary tumors (Fig. 3).

**Fig. 4:**
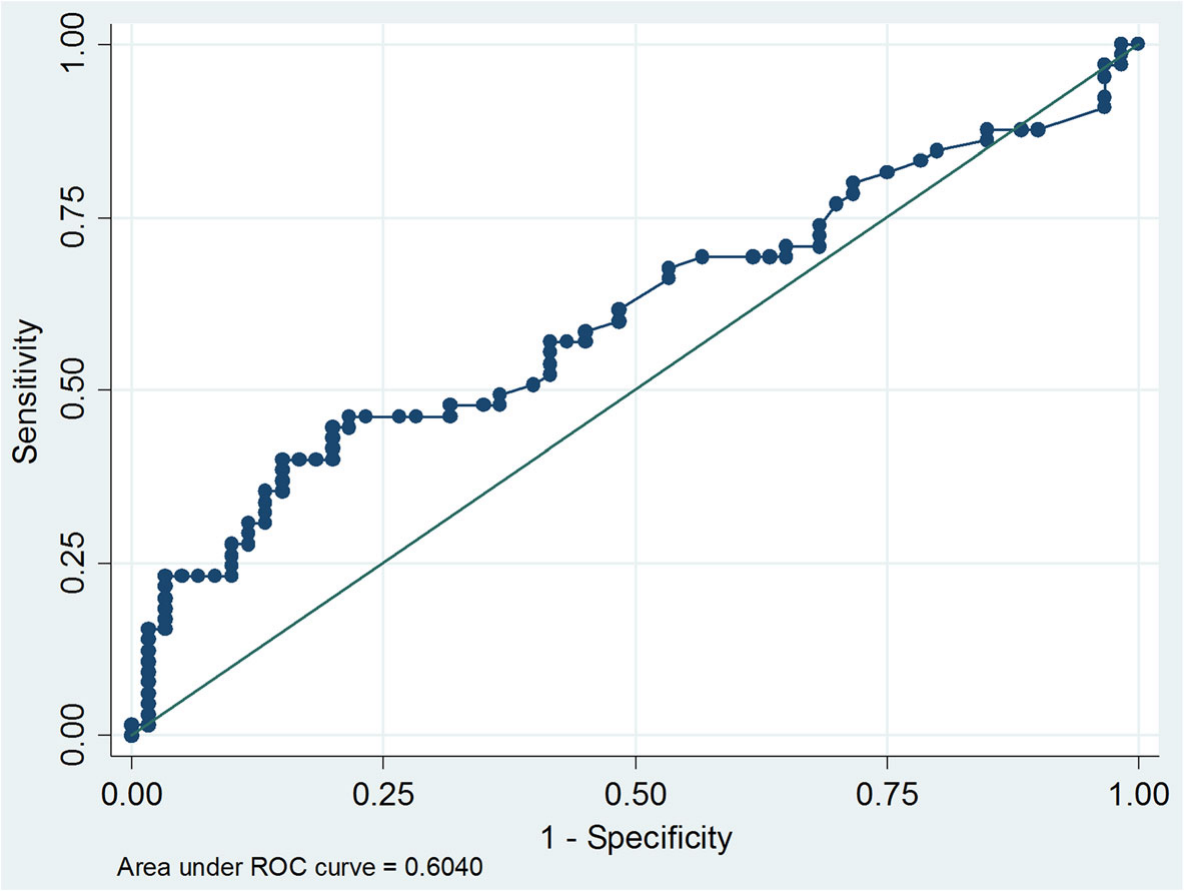
Receiver operating characteristic curves for predicting tumor recurrence

**Fig. 5:**
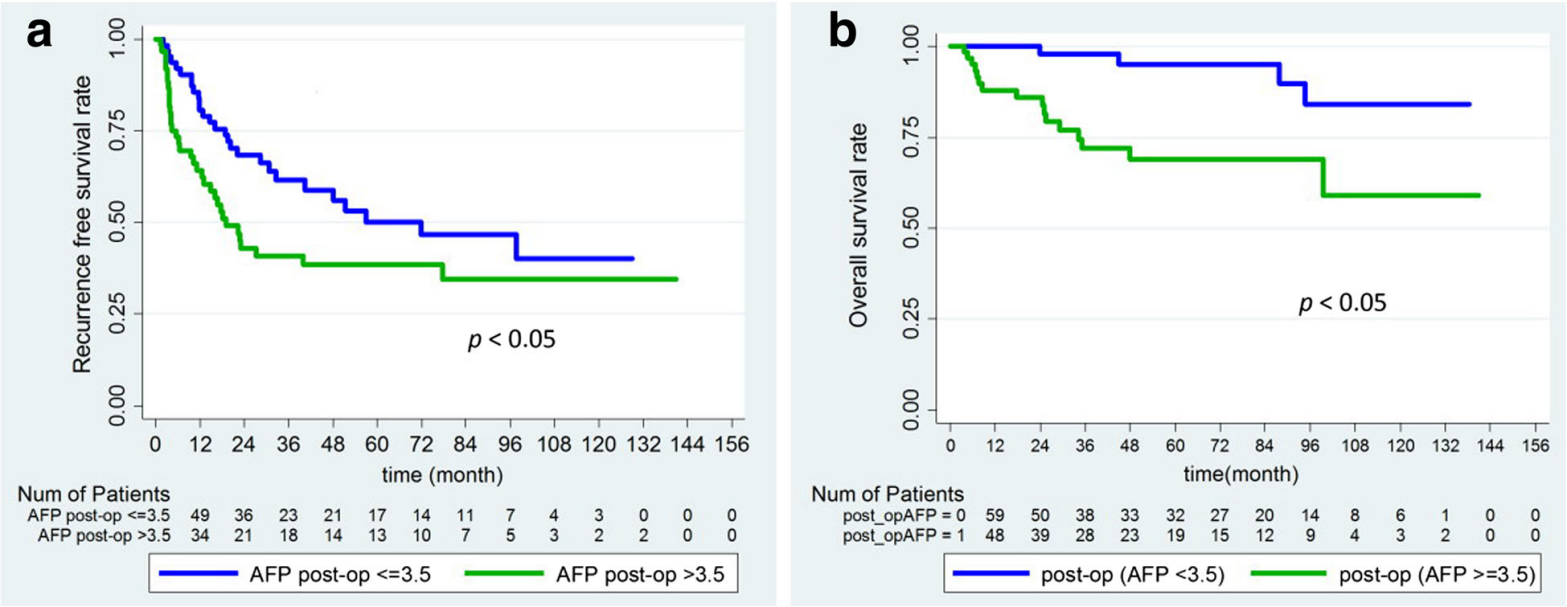
Kaplan-Meier survival analysis of patients with post-operative AFP < 3.5 and post-operative AFP ≥ 3.5 groups. **a**, recurrence-free survival; **b**, overall survival

In addition, post-operative AFP was the risk factor of recurrence. Comparison of the patients between high and low post-operative AFP groups. As the first step, the cut-off value for post-AFP was determined by receiver operating characteristic (ROC) curve analysis as shown in Fig. 4. The area under ROC curve was 0.604. The post-operative AFP value 3.5 ng/mL was considered as the optimal cut-off value because of its highest index; the sensitivity and specificity were 56.9 and 58.3%, respectively. The Kaplan-Meier analysis curves for RFS and OS of patients with post-operative AFP level > 3.5 ng/mL had poorer overall and recurrence free survival when compared with post-operative AFP level ≤ 3.5 ng/mL(Fig. 5).

### Outcomes correlation stratified by antiviral treatment in solitary and multiple tumor

**Fig. 6:**
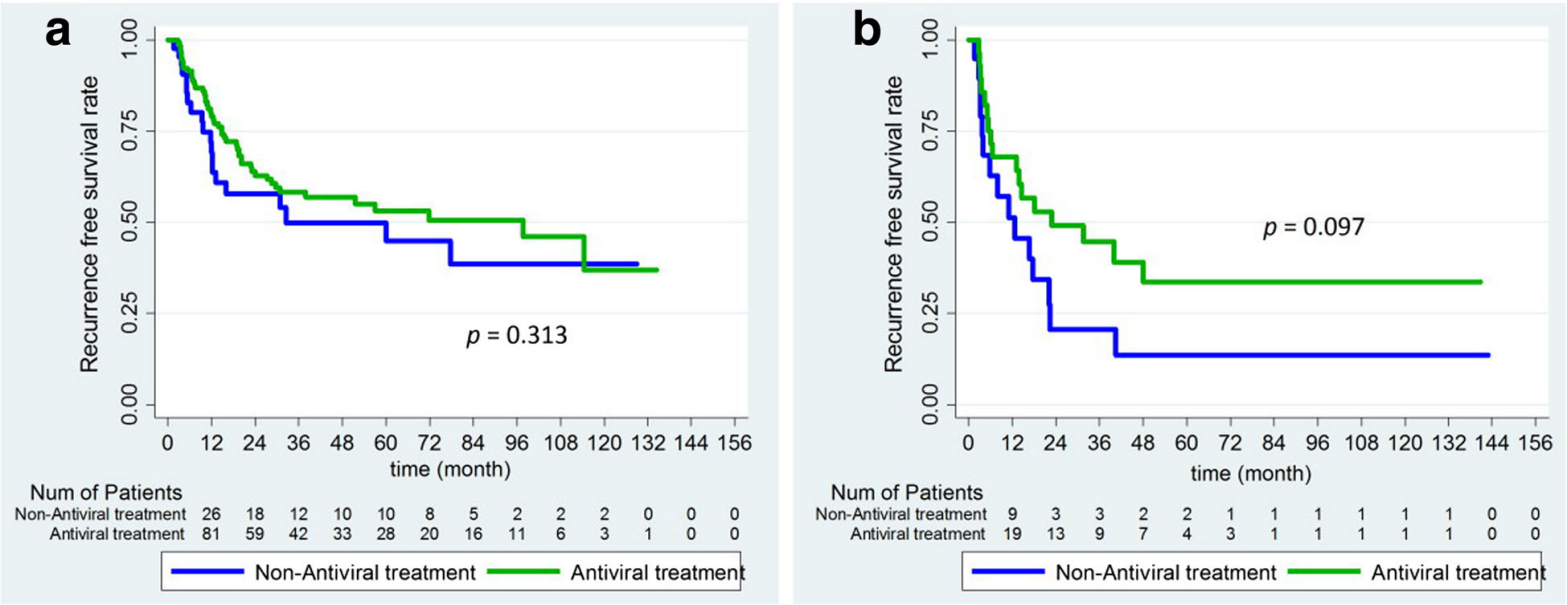
Kaplan-Meier survival analysis of patients with or without antiviral treatment according to the number of tumor. **a**, solitary tumor; **b**, multiple tumor

The Kaplan-Meier analysis curves for RFS of patients who had soliltary and multiple tumor with or without antiviral treatment (Fig. 6). The RFS in the solitary and multiple tumor groups were not significantly difference with antiviral compared with non-antiviral treatment.

## Discussion

Chronic HBV infection is a major risk factor for the development of HCC, especially in Southeast Asia [26]. The pathogenesis of HBV-induced HCC is complex and involves both direct and indirect mechanisms. The immune response against HBV-infected hepatocytes triggers inflammation and leads to sustained necrosis [12]. Recent work has suggested a role for platelets in promoting liver infiltration of cytotoxic T lymphocytes and non-virus-specific inflammatory cells in the pathogenesis of HCC in a HBV transgenic mouse model [20, 27]. In addition, biomarkers such as AFP and inflammatory mediators have been reported to affect the prognosis of HBV-related HCC patients [15, 18, 19, 28–32], although the results are controversial.

In our study, we found that post-operative serum AFP levels and the presence of multiple tumors are predictors of poor prognosis for HBV-related HCC following hepatic resection. AFP is a large glycoprotein produced by the yolk sac and fetal liver. AFP is present in large quantities during gestation and is generally repressed in healthy adults; however, it is re-expressed in a variety of tumors [33, 34]. Several studies have reported correlations between AFP levels and the prognosis of HBV-related HCC patients after curative resection, but most of them measured only preoperative AFP levels and the prognostic impact of AFP levels following hepatic resection was unclear [15, 35–40]. In other studies, post-operative AFP levels were shown to correlate with the prognosis of HCC patients, but the populations in those studies were heterogenous and included both HBV-positive and -negative patients [41–47]. Here, we show for the first time that the post-operative serum AFP level is an independent prognostic factor for survival in HBV-related HCC patients following curative resection. Our results are consistent with a study by Shen et al., who reported that a ≤ 50% difference between pre- and post-operative serum AFP was predictive of poor disease-free and overall survival after hepatectomy in HCC patients, 89.3% of whom had HBV-related HCC [41]. Allard et al. reported that a post-resection AFP level of > 15 ng/mL was a poor predictor of outcome for cirrhotic HCC patients with preoperative AFP levels of > 15 ng/ml [43]. Similarly, Zhang et al. reported that high serum AFP and alpha-fetoprotein-L3 (AFP-L3) levels before and after hepatectomy predicted poor survival [46].

Several potential mechanisms could account for the association between high post-operative serum AFP levels and survival outcome in HBV-related HCC patients. First, although AFP is not present at elevated levels in early-stage HCC and is thus a poor diagnostic biomarker [29, 48, 49], high serum AFP levels may reflect an increasing disease burden due to extrahepatic metastasis, advanced stage, large tumor size, and/or portal vein thrombosis [50]. Ogden et al. and Sung et al. reported that the HBV viral protein HBx dysregulates p53-mediated AFP expression through direct binding to p53, and high HBV integration into the host genome correlated with high serum AFP levels [51, 52]. Moreover, Silva et al. reported that baseline serum AFP levels were higher in HCC patients with more advanced disease and could predict their overall survival, regardless of treatment. Therefore, the patients with high post-operative serum AFP levels in our study may have had occult intra- or extrahepatic metastasis [48]. In addition, high serum AFP may be a marker of liver inflammation in patients with chronic liver disorders [10, 12, 50]. Sitia et al. reported that inflammation was a key event in HCC carcinogenesis in HBV transgenic mice and was promoted by lymphocyte infiltration and platelet aggregation [21]. Therefore, ongoing inflammation in patients with high serum AFP could facilitate hepatic carcinogenesis.

In this study, we also found that the presence of multiple HCC tumors is a predictor of recurrence after initial hepatic resection. This is consistent with previous studies showing that multiple tumors is one of the most significant risk factors of early tumor recurrence and poor outcome in HBV-related HCC patients [53–55]. Intrahepatic recurrence is also associated with survival of HCC patients [56]. In agreement with these observations, our multivariate analysis identified tumor recurrence as an independent predictor of poorer overall survival. Park et al. reported that multiple tumors resulting from intrahepatic metastasis was a strong predictor of early multinodular intrahepatic recurrence in HCC patients following hepatic resection [54]. Hao et al. reported that the presence multiple tumors was significantly associated with intrahepatic metastasis recurrence in HBV-related HCC patients, whereas liver cirrhosis and hepatic inflammation activity were associated with multi-centric recurrence [57]. These authors concluded that intrahepatic and multi-centric metastasis recurrence were mainly caused by tumor-related factors and patient-related factors, respectively [57]. Our results showing that patients with solitary and multiple tumors had significantly different recurrence-free and overall survival rates are consistent with this study. We hypothesize that our patients with multiple tumors may have had intrahepatic metastasis and multi-focal occult tumors.

We examined a number of inflammatory markers, including neutrophil-to-lymphocyte ratio, platelet-to-lymphocyte ratio, and prognostic nutritional index, in our patient cohort and found that none of them predicted survival. Antiplatelet therapy also was not a prognostic indicator, although 16 of the 18 patients who received this therapy survived. The small sample population may explain why this finding was not statistically significant. The benefit of antiplatelet therapy in HBV-related HCC patients has been investigated in two large retrospective studies [23, 58]. In a study of Taiwanese patients, Lee et al. found that antiplatelet therapy, including aspirin or clopidogrel, was associated with better recurrence-free survival and overall survival following hepatic resection. However, antiplatelet use significantly increased the risk of upper gastrointestinal bleeding in that study. Lee et al. found that antiplatelet therapy reduced the risk of HCC in South Korean patients whose chronic HBV infection had been effectively suppressed. However, clopidogrel alone with aspirin was found to increase the risk of bleeding [58]. Large-scale prospective studies are clearly needed to unequivocally establish the benefits and risk of complications from antiplatelet therapy.

This study has several limitations. First, it was retrospective in nature. Second, AFP levels in patients with HBV infection could be affected by non-malignancy-related factors such as liver cirrhosis, acute hepatitis, and chronic liver disease [50]. In this study, we included HBV-infected patients with and without cirrhosis and there are seven patients enrolled in the study were co-infected with HBV and HCV. The etiology of HCC among those patients may not due to the chronic HBV infection. Third, there are a number of studies indicating that biomarkers such as protein induced by vitamin K absence-II [32], des-gamma carboxy prothrombin [39], and AFP-L3 [59] may be more accurate prognostic biomarkers than AFP level. However, these tumor markers are not currently measured at our hospital. Fourth, some patients especially in the early period of the study were not treated with anti-viral drugs. Fifth, the patients who neoadjuvant therapy were performed, the AFP level and inflammatory marker levels could be affected. Sixth, the number of death population could be slightly lower than actual due to there are some patients who had recurrence disease have loss to follow-up. Seventh, lamivudine is an anti-HBV drug of modest antiviral effect with low barrier of drug resistance and is no longer suggested by American Association for the Study of Liver Diseases and European Association of the Study of the Liver as a first-line antiviral option [60, 61]. The proportion of patients with lamivudine treatment in this study was relatively high, which may lead to underestimation of the protective effect of antiviral treatment on HBV related HCC recurrence.

## Conclusions

Post-operative serum alpha-fetoprotein level and multiple tumors, but not inflammatory indices, platelet counts, or antiplatelet therapy, were found to be risk factors of poor prognosis for HBV-related HCC patients following hepatectomy. Prospective studies will be required to clarify the role of platelets in the disease and the benefits of antiplatelet therapy in this patient group. Our results indicate that patients with multiple tumors and high post-operative serum alpha-fetoprotein level should be monitored carefully following hepatic resection.

## Abbreviations

AFP: Alpha-fetoprotein
AFP-L3: Alpha-fetoprotein L3
CI: Confidence intervals
CT: Computed tomography
HBV: Hepatitis B virus
HCC: Hepatocellular carcinoma
HR: Hazard ratio
ICG-R15: Indocyanine green retention at 15 min
MRI: Magnetic resonance imaging
